# Regulation of lipid synthesis by the RNA helicase Mov10 controls Wnt5a production

**DOI:** 10.1038/oncsis.2015.15

**Published:** 2015-06-01

**Authors:** W Wang, N Snyder, A J Worth, I A Blair, E S Witze

**Affiliations:** 1 Department of Cancer Biology, University of Pennsylvania, Philadelphia, PA, USA; 2 Abramson Family Cancer Research Institute, University of Pennsylvania, Philadelphia, PA, USA; 3 Perelman School of Medicine, University of Pennsylvania, Philadelphia, PA, Philadelphia, PA, USA; 4 Department of Pharmacology, University of Pennsylvania, Philadelphia, PA, USA; 5 Institute for Translational Medicine and Therapeutics, University of Pennsylvania, Philadelphia, PA, USA; 6 AJ Drexel Autism Institute, Drexel University, Philadelphia, PA, USA

## Abstract

Expression of the Wnt ligand Wnt5a is frequently elevated in melanoma and is thought to be a critical regulator of cell movement during metastasis. However, the mechanisms regulating its expression are unknown. We find that the level of secreted Wnt5a varies by as much as 10-fold between cell lines and correlates more strongly with invasion than total cellular levels. Our results indicate that the RNA helicase Mov10 plays a role in Wnt5a synthesis and secretion. Inhibition of Mov10 increases secreted Wnt5a levels in melanoma cells by increasing Wnt5a synthesis and acylation. This is achieved by increasing fatty acid synthase (FASN) and stearoyl-CoA desaturase expression, leading to elevated levels of palmitoleoyl-CoA, required for Wnt ligand lipid modification and secretion. Melanoma tumors exhibit reduced expression of Mov10 compared with benign nevi and Mov10 levels inversely correlate with FASN levels in primary tumors. These results reveal a previously unappreciated role for aberrant lipid metabolism in regulating Wnt5a signaling that may be a critical step in melanoma progression.

## Introduction

Wnt5a is a member of the Wnt family of secreted ligands that signals independently of β-catenin-mediated transcription.^1, 2^ Wnt5a is strongly implicated in promoting metastatic behavior in melanoma and other types of cancer and is thought to function as an autocrine signaling factor to promote cell motility.^3, 4, 5, 6, 7, 8, 9, 10, 11^ It has been demonstrated that Wnt5a protein levels are elevated in late-stage melanoma patient samples, and increased Wnt5a expression increases cell motility, invasion and polarization of the cytoskeleton in melanoma cell lines.^9, 10, 11^ Wnt5a interacts with the receptor tyrosine kinase Ror2 and downstream signaling requires the cytosolic adaptor protein Disheveled.^12, 13^ Inhibition of the Wnt5a receptor Ror2 in melanoma blocks lung colonization in mice, demonstrating a requirement for the Wnt5a pathway in extravasation and colonization during metastasis.^14^ Although Wnt5a levels increase with melanoma progression, the molecular events that promote Wnt5a expression in melanoma are largely unknown.

Wnt ligand secretion requires acylation of serine residues with the unsaturated fatty acid palmitoleic acid mediated by the membrane-bound *O*-acyl transferase Porcupine (PORCN).^15, 16, 17, 18, 19^ Recent studies also show that stearoyl-CoA desaturase (SCD) enzymes that convert palmitate to palmitoleic acid, the lipid donor for Wnt, are also required for Wnt acylation and secretion.^20, 21^ Although it is apparent that the enzymes PORCN and SCD are required for wild-type Wnt ligand secretion, studies of the regulation of Wnt5a secretion in cancer and the effect on disease progression are lacking.

Mov10 is an RNA helicase that regulates mRNA stability and translation, playing critical roles in embryonic development and neuronal function. Mov10 translocates 5′ to 3′ along the mRNA 3′-untranslated region (UTR) and decreases the half-life of mRNA targets.^22^ Mov10 also interacts with Argonaute2 in the RISC (RNA-induced silencing complex) and is required for microRNA (miRNA)-mediated inhibition in Drosophila and mammalian cells.^23, 24^ Mov10 is not required for miRNA processing, but is critical for efficient RNA-induced silencing.^23^ Mov10 has also been shown to recruit eIF6 to mRNA. eIF6 is an initiation factor that inhibits the formation of the 80S ribosomal complex and blocks protein translation.^25, 26^

Data regarding the biological function of Mov10 are limited. In Drosophila Mov10 mutants (*Armitage*) display defects in embryonic dorsal/ventral patterning and Mov10 is required for the translational silencing of the *oskar* mRNA.^27^ In neuronal synapses Mov10 is degraded in response to ion channel activation relieving translation inhibition.^28^ Hypomorphic alleles of Mov10 result in defects in protein expression at synapses and long-term memory in Drosophila.^29^ These studies have demonstrated that specific cell types and developmental stages can be more sensitive to reduced Mov10 function.

Our studies reveal a previously unreported role for Mov10 in regulating the Wnt5a signaling pathway during melanoma progression. We show Mov10 protein levels are reduced in melanoma tumor samples stained by immunohistochemistry when compared with benign nevi. Reduction of Mov10 expression by short hairpin RNA (shRNA) increases the level of lipid modified and secreted Wnt5a in melanoma cell lines. Cells expressing Mov10 shRNA also show increased cell invasion in three-dimensional collagen that is blocked by inhibiting the Wnt5a receptor Ror2 by shRNA. The increased level of secreted Wnt5a is partly driven by elevated synthesis of the unsaturated lipid donor palmitoleoyl-CoA resulting from increased expression of FASN and SCD. Finally, tumors that express low levels of Mov10 express higher levels of FASN, providing correlative evidence for Mov10-regulated lipid metabolism in cancer.

## Results

### Levels of Wnt5a secretion correlates with cell invasion in melanoma cells

**Figure 1:**
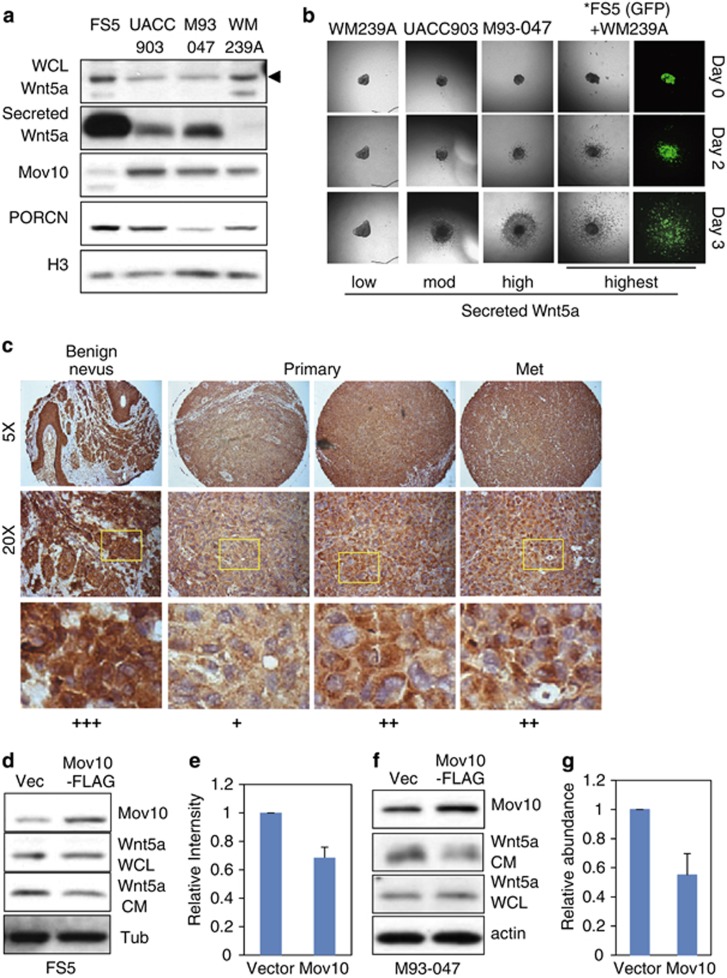
Levels of secreted Wnt5a correlate with cell invasion. (**a**) Protein levels of total Wnt5a (arrowhead), secreted Wnt5a, Mov10 and PORCN in different metastatic melanoma cell lines. Histone H3 was used as a loading control. (**b**) Spheroid invasion assay on the cell lines in (**a**) measures cell invasion into collagen. *FS5 cells failed to form spheroids and were therefore mixed with WM239A for the invasion assay. (**c**) Expression of Mov10 in benign nevi, malignant melanoma and metastatic melanoma samples at × 5 and × 20 magnification. Samples were scored on × 20 magnification as high (+++), medium (++) and low (+) Mov10 staining. (**d**, **f**) Secretion of Wnt5a in conditioned media (CM) from FS5 and M93-047 cells ectopically expressing Mov10-FLAG. Cells were counted after collection of conditioned media and samples were normalized to the cell number. (**e**, **g**) Quantification of western blots in (**d**, **f**). M93-047 cells are an average of three experiments (*P*=0.006, Student's *t*-test of ectopic Mov10-FLAG-expressing cells compared with vector control). FS5 cells are an average of two experiments. Error bars denote s.d.

To examine the correlation between Wnt5a expression and cell invasion the level of Wnt5a protein in whole-cell lysates from multiple melanoma cell lines was determined by immunoblotting. The FS5 and WM239A cells expressed higher levels of Wnt5a than M93-047 and UACC903 cells (Figure 1a). We expected to see greater invasion in the higher Wnt5a-expressing lines if Wnt5a is a significant factor in determining invasiveness; however, we found little correlation between the invasiveness of the cell line and the level of Wnt5a measured in the cell lysates in a three-dimensional collagen invasion assay (Figures 1a and b). As Wnt5a is a secreted ligand, we asked whether invasion may instead correlate with the levels of Wnt5a secreted by the different cell lines. In contrast to the total Wnt5a levels in the cell lysates the levels of Wn5a secreted into the media differed by as much as 10-fold between the FS5 and WM239A lines (Figure 1a). The melanoma cell lines WM239A, UACC903, M93-047 and FS5 were found to secrete increasing levels of Wnt5a respectively. The level of secreted Wnt5a strongly correlated with the degree of cell invasion in collagen, implicating Wnt5a secretion as a significant factor for determining cell invasion *in vitro* (Figure 1b).

To determine whether Wnt5a protein levels correlated with mRNA expression the levels of Wnt5a mRNA were determined by quantitative real-time PCR in the different cell lines. The quantitative real-time PCR analysis revealed that Wnt5a mRNA levels did not correlate with total Wnt5a protein levels (secreted+whole-cell lysate), suggesting that Wnt5a is regulated through a post-transcriptional mechanism (Supplementary Figure S1). As Wnt5a expression has been reported to be regulated by miRNAs in specific cellular contexts including prostate cancer, we hypothesized that regulation of protein translation or mRNA stability could be a mechanism for determining Wnt5a production levels in melanoma.^30^

### Decreased Mov10 expression promotes cell invasion and Wnt5a secretion

We looked at factors associated with the RISC pathway that may regulate Wnt5a levels during melanoma progression. The RNA helicase Mov10 interacts with Argonaute to mediate miRNA-mediated repression of translation and mRNA turnover.^23, 24^ Searching the data published by Gregersen *et al.*^22^ using PAR-CLIP (photoactivatable ribonucleoside-enhanced crosslinking and immunoprecipitation) revealed that Mov10 interacts with Wnt5a mRNA transcripts and mutations in the helicase domains abrogated the interaction. We asked whether Mov10 may be involved in Wnt5a expression and secretion. In the cell lines examined the high Wnt5a secreting line FS5 had markedly lower levels of Mov10 protein relative to the other cell lines (Figure 1a).

To determine the levels of Mov10 protein in melanoma patient samples, tissue microarrays containing benign nevi and malignant melanoma were stained by immunohistochemistry to detect Mov10 protein. The samples were scored blindly for Mov10 staining and ranked as high (+++), medium (++) and low (+) Mov10 expression levels (Figure 1c and Table 1). The Mov10 protein levels in benign nevi were found to be higher than levels observed in the malignant melanoma (Figure 1c and Table 1). The melanoma samples that were derived from secondary tumors expressed higher levels of Mov10 than the primary tumor samples, but were lower than the benign tumors (Figure 1c and Table 1).

**Figure 2:**
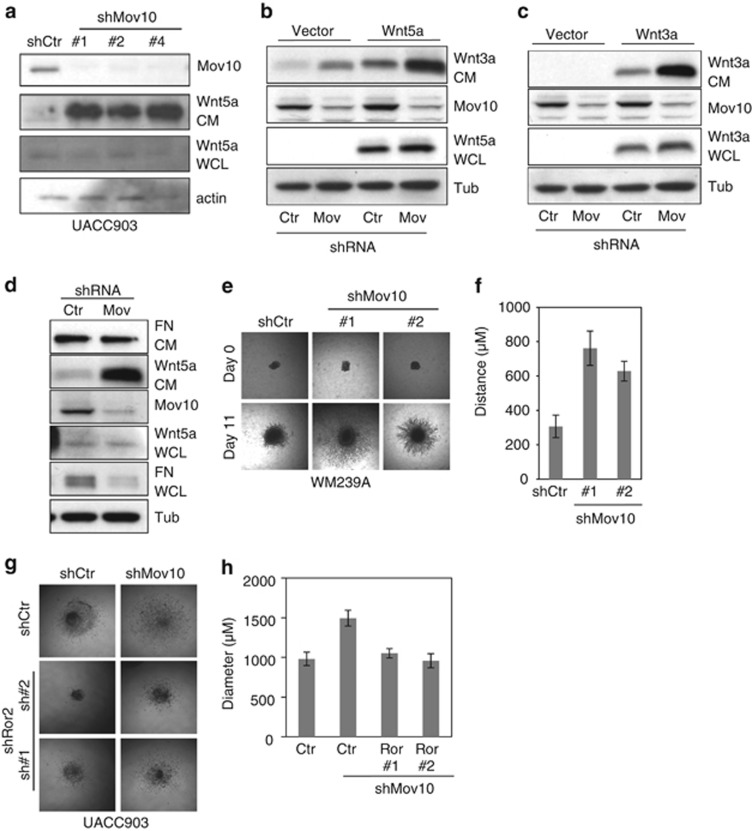
Inhibition of Mov10 increases Wnt5a secretion and Ror2-dependent cell invasion. (**a**) Wnt5a secretion in conditioned media (CM) from UACC903 melanoma cells compared with whole-cell lysate (WCL) from control and Mov10 shRNA-expressing cells. (**b**) Wnt5a secretion in UACC903 cells expressing a Wnt5a transgene. (**c**) Wnt3a secretion in UACC903 cells expressing a Wnt3a transgene. (**d**) Secretion of fibronectin (FN) from UACC903 cells expressing control and Mov10 shRNA. (**e**) Collagen invasion of WM239A melanoma cells expressing two Mov10 shRNA constructs. (**f**) Quantification of invasion assay in (**a**) Student's *t*-test, compared with shControl, shMov10-1 *P*=2.29 × 10^−11^, shMov10-2 *P*=3.95 × 10^−10^. (**g**) Collagen invasion assay of cells expressing Ror2 shRNA and Mov10 shRNA (see Supplementary Figure S2). (**h**) Quantification of invasion assay in (**d**). Student's *t*-test, shMov10+shCtr vs shCtr *P*=3.07 × 10^−11^, shMov10+shRor2-1 vs shMov10+shCtr *P*=9.02 × 10^−10^, shMov10+shRor2-2 vs shMov10+shCtr *P*=1.42 × 10^−11^. Error bars indicate s.d.

We next asked whether Mov10 is sufficient or necessary for suppressing the level of secreted Wnt5a. Mov10-FLAG was ectopically expressed in the high Wnt5a secreting melanoma cell lines FS5 and M93-047. Although the ectopic expression of Mov10-FLAG was modest, the level of secreted Wnt5a was significantly reduced, whereas the level of Wnt5a in the whole-cell lysate was unchanged (Figures 1d–g). Next, Mov10 expression was inhibited by shRNA in the moderate Wnt5a secreting melanoma cell line UACC903. Inhibition of Mov10 did not significantly increase Wnt5a levels in whole-cell lysate, but the levels of secreted Wnt5a increased dramatically in cells expressing three separate short hairpins targeting Mov10 (Figure 2a). Although an increase in Wnt5a protein was not observed in the cell lysates, the high levels of secreted Wnt5a in the media indicate there is a coordinated increase in Wnt5a synthesis and secretion upon inhibition of Mov10.

To confirm that the increase in Wnt5a production caused by inhibition of Mov10 was not due to an increase in Wnt5a transcription, the Wnt5a complementary DNA was stably expressed by the phosphoglycerate kinase (PGK) promoter in shControl and shMov10 cells. Both Wnt5a and Wnt3a secretion was increased upon inhibition of Mov10 in spite of the absence of 5′ and 3′-UTRs (Figure 2b). A similar response was seen in cells ectopically expressing Wnt3a. These results indicate the increase in Wnt5a secretion was not caused by Mov10 binding to Wnt5a mRNA as the Mov10-binding sites are in the 5′ and 3′-UTRs. To determine whether Mov10 shRNA increases general cell secretion, the conditioned media containing Wnt5a were probed for the secreted protein fibronectin. The level of secreted Wnt5a increased in the shMov10 cells, whereas there was no detectable change in fibronectin in the media (Figure 2d).

If inhibition of Mov10 is sufficient to increase levels of secreted Wnt5a, it should also increase cell invasion. The low Wnt5a secreting WM239A cells expressing Mov10 shRNA displayed significantly higher collagen invasion, and the effect was observed with two short hairpins (Figures 2e and f). To address whether the increase in invasion is caused by Wnt5a signaling activity the Wnt5a receptor Ror2 was inhibited by two separate short hairpins in control and Mov10 shRNA-expressing UACC903 cells (Supplementary Figure S2). When Ror2 expression was inhibited in cells expressing Mov10 shRNA, the invasion in collagen was markedly reduced, although not completely blocked (Figures 2g and h).

### Wnt5a acylation and SCD are increased by Mov10 inhibition

**Figure 3:**
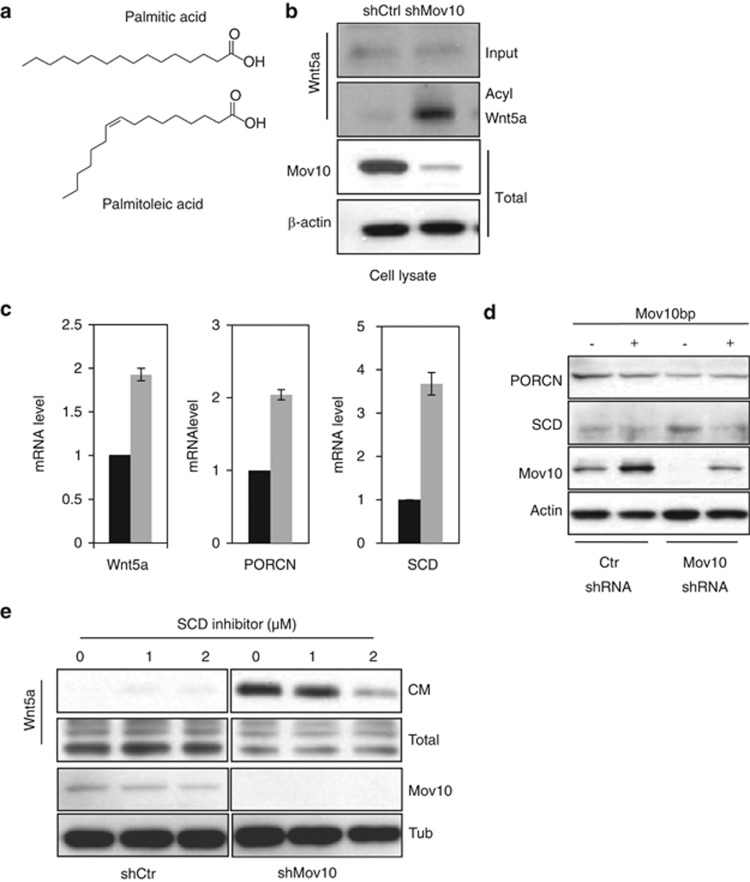
Inhibition of Mov10 increases Wnt5a acylation and requires SCD for secretion. (**a**) Structures of palmitic acid and palmitoleic acid. (**b**) Levels of lipid-modified (acyl) Wnt5a in cell lysates from metabolically labeled Mov10 shRNA- and control shRNA-expressing cells. Input is the Wnt5a level in the cell lysate before pull down. (**c**) Wnt5a PORCN and SCD mRNA levels in Mov10 shRNA (gray bars) and control shRNA expressing (black bars). Levels of mRNA are relative to L32 mRNA. (**d**) PORCN and SCD protein levels in UACC903 cells expressing Mov10 shRNA, control shRNA or Mov10 shRNA and expressing an shRNA-resistant bypass Mov10 (Mov10bp). (**e**) Wnt5a secretion in UACC903 cells expressing Mov10 shRNA followed by treatment with the SCD inhibitor CAY10566 (1–2 μM) or dimethyl sulfoxide (DMSO) control.

*O*-acylation of Wnt5a with palmitoleic acid on serine is required for Wnt5a secretion (Figure 3a).^17, 19^ To determine whether Mov10 inhibition increases Wnt5a acylation, UACC903 cells were metabolically labeled with palmitic acid azide (see Materials and methods). Inhibition of Mov10 in UACC903 cells increased the level of acylated Wnt5a in the cell lysate by as much as 10-fold compared with control shRNA, whereas the total level of Wnt5a in the lysate (input) was unchanged (Figure 3b). This indicates that Mov10 inhibits Wnt5a acylation that could be mediated by decreased levels or activity of PORCN. Of the cell lines examined in this study, the levels of Wnt5a secretion did not correlate with the PORCN protein levels (Figure 1a). Furthermore, although inhibition of Mov10 by shRNA increased levels of PORCN mRNA, the protein levels remained unchanged (Figures 3c and d). We confirmed the PORCN antibody is specific to the PORCN protein as three separate shRNA hairpins decrease levels of PORCN protein (Supplementary Figure S3). This suggests that Mov10 shRNA increases Wnt5a acylation by a mechanism that does not require increased PORCN expression levels.

Recent studies indicate that enzymes involved in the synthesis of the fatty acid palmitoleic acid are required for Wnt ligand lipid modification and secretion.^20, 21^ We therefore asked whether the synthesis of palmitoleic acid is affected by Mov10 shRNA. Conversion of palmitic acid (16:0) to the unsaturated palmitoleic acid (16:1) is mediated by the stearoyl-CoA desaturase enzymes SCD and SCD5. SCD is ubiquitously expressed throughout tissues, whereas SCD5 is expressed mainly in the brain.^31^ We chose to focus our studies on the more widely expressed SCD. The level of SCD mRNA and protein was higher in UACC903 cells expressing Mov10 shRNA compared with control cells, and expression of a shRNA-resistant Mov10 decreased SCD protein levels down to control shRNA levels (Figures 3c and d). To determine whether the increased Wnt5a secretion required SCD, shControl and shMov10-expressing cells were treated with the SCD inhibitor CAY10566. The increase in Wnt5a secretion caused by Mov10 shRNA was inhibited by CAY10566 dose dependently, demonstrating the requirement of SCD activity for the increase in Wnt5a secretion caused by Mov10 inhibition (Figure 3e).

### Mov10 shRNA increases palmitoleoyl-CoA and palmitoyl-CoA levels

**Figure 4:**
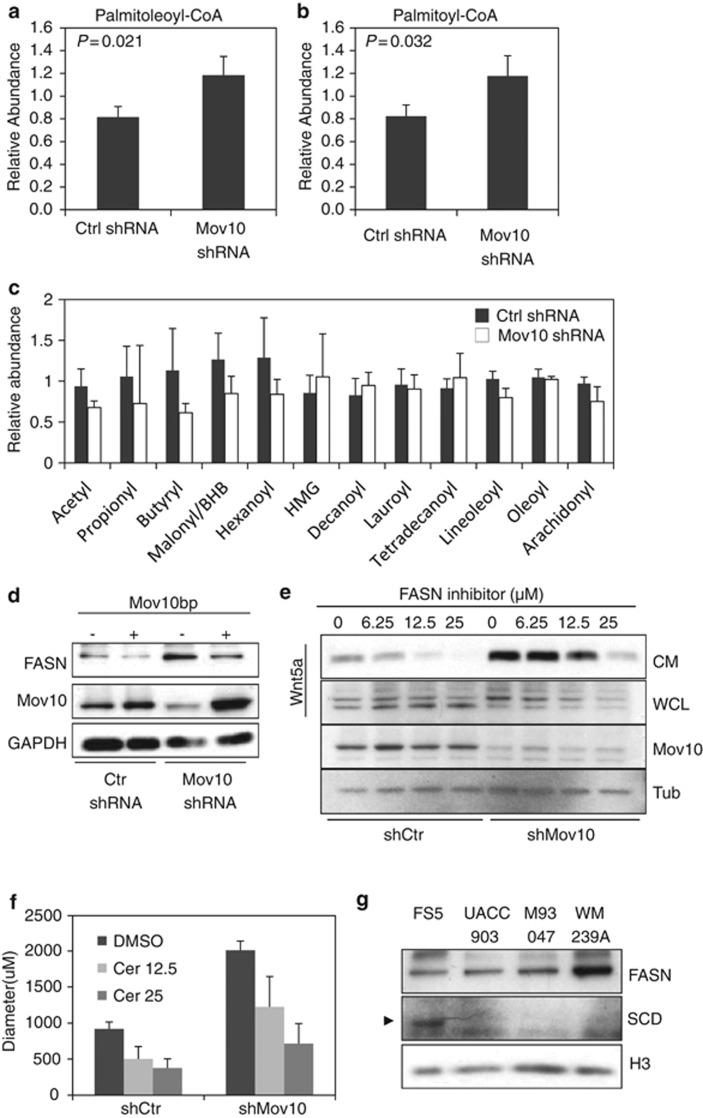
Mov10 inhibition increases levels of palmitoyl-CoA and palmitoleoyl-CoA. (**a**) Levels of palmitoleoyl-CoA in Mov10 shRNA-expressing UACC903 melanoma cells determined by mass spectrometry. (**b**) Increased levels of palmitoyl-CoA in Mov10 shRNA-expressing melanoma cells. (**c**) Profile of acyl-CoA species in control (gray bars) and Mov10 shRNA (white bars)-expressing cells determined by mass spectrometry. (**d**) FASN protein levels in UACC903 cells expressing Mov10 shRNA, control shRNA or Mov10 shRNA and expressing an shRNA-resistant bypass Mov10 (Mov10bp). (**e**) Wnt5a secretion in UACC903 melanoma cells expressing Mov10 shRNA treated with the FASN inhibitor cerulenin. Error bars represent the s.d. of four biological replicates. (**f**) FASN inhibitor cerulenin blocks invasion of UAC903 melanoma cells (dimethyl sulfoxide (DMSO) *n*=7, cerulenin 12.5 μM *n*=10, cerulenin 25 μM *n*=10, *P*<1 × 10^−7^). (**g**) Melanoma cell lines shown in Figure 1a probed for FASN and SCD. *P*-values determined by Student's *t*-test.

To determine whether the increase in SCD mRNA observed in shMov10-expressing cells also leads to increased levels of palmitoleoyl-CoA, stable isotope dilution liquid chromatography–mass spectrometry was used to directly measure the levels of palmitoleoyl-CoA in control shRNA and Mov10 shRNA-expressing UACC903 cells.^32^ The relative abundance of palmitoleoyl-CoA significantly increased in Mov10 shRNA cells compared with shRNA control (Figure 4a). If the conversion of palmitoyl-CoA to palmitoleoyl-CoA is increased by Mov10 shRNA, we might expect to see a coordinated decrease in palmitoyl-CoA. Unexpectedly, the level of palmitoyl-CoA also increased in Mov10 shRNA-expressing cells compared with the shRNA control cells (36.45±5.51 vs 25.50±3.07; Student's *t*-test, *P*=0.032) suggesting Mov10 shRNA also increases levels of enzymes involved in synthesis of palmitate (Figure 4b). When other acyl-CoA groups were profiled by mass spectrometry, most acyl-CoA forms were either unchanged or slightly reduced in cells expressing Mov10 shRNA, indicating the effect of Mov10 inhibition on lipid synthesis is specific to palmitoyl-CoA and palmitoleoyl-CoA pathways (Figure 4c). One pathway for the *de novo* synthesis of palmitate is through the FASN. FASN is expressed at low levels in most cells but is elevated in many types of cancer including melanoma.^33, 34, 35, 36, 37, 38^ Inhibition of Mov10 by shRNA caused an increase in the levels of FASN protein that was suppressed by expression of a bypass Mov10 containing shRNA-resistant mutations (Figure 4d). We found inhibiting FASN with the small-molecule inhibitor cerulenin was sufficient to block the increase in secreted Wnt5a induced by Mov10 shRNA (Figure 4e). Inhibition of FASN with cerulenin also decreases invasion of UACC903 cells in a dose-dependent manner (Figure 4f and Supplementary Figure S4). The levels of FASN and SCD were examined in the cell lines in Figure 1a. The highest Wnt5a secreting line FS5 expressed the highest level of SCD consistent with the expression levels of SCD being critical for Wnt5a secretion (Figure 4g). Unexpectedly, highest level of FASN was expressed in the lowest secreting cell line WM239a expressed (Figure 4g). This may indicate that in cultured cells FASN is not rate limiting for Wnt5a secretion.

### Mov10 interacts with Wnt5a, SCD and FASN mRNA transcripts

**Figure 5:**
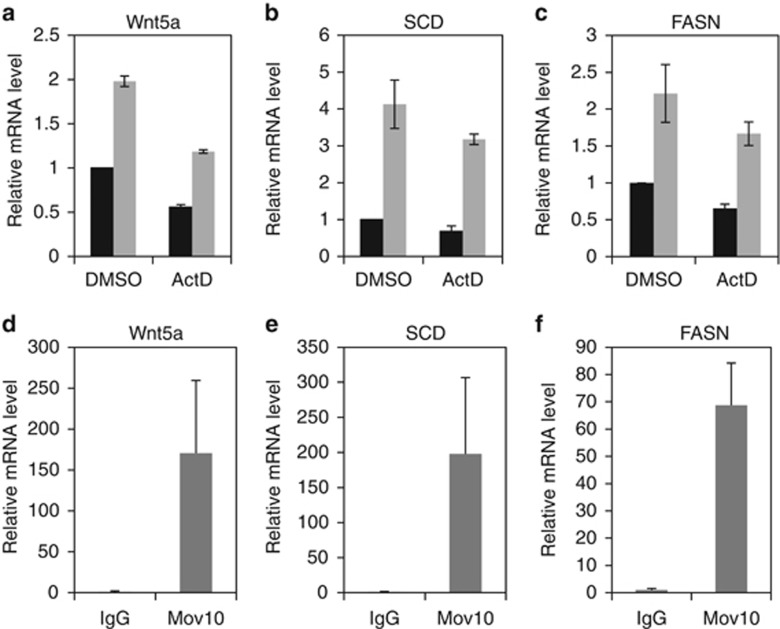
Mov10 directly regulates mRNA levels post transcriptionally. (**a–c**) FASN, SCD and Wnt5a levels in UACC903 cells expressing Mov10 shRNA (gray bars) or control shRNA (black bars) after treatment with actinomycin D (5 μg/ml) for 8 h. (**d–f**) Wnt5a, SCD and FASN mRNA detected in anti-Mov10 immunoprecipitation in UACC903 cells compared with control IgG.

To determine whether the increase in Wnt5a, FASN and SCD mRNA in response to Mov10 shRNA is transcriptionally independent, mRNA levels were determined in cells treated with actinomycin D (5 μg/ml) for 8 h to inhibit mRNA transcription before harvesting cell lysates. The levels of Wnt5a, FASN and SCD mRNA in Mov10 shRNA-expressing cells after actinomycin D treatment were elevated between two- and five-fold compared with the control shRNA cells, similar to dimethyl sulfoxide control-treated cells (Figures 5a–c). The transcriptional independence of the increased mRNA levels is consistent with the function of Mov10 regulating mRNA stability or indirectly increasing the mRNA levels by increasing the rate of translation.^39^

Mov10 is an RNA helicase that inhibits mRNA translation or stability through either RISC or by nonsense-mediated mRNA decay.^22^ Therefore, the inhibition of the Wnt5a, SCD and FASN expression would be expected to involve interaction between Mov10 and the putative mRNA targets. To determine whether Mov10 interacts with Wnt5a, SCD or FASN mRNA in melanoma cells, Mov10 was immunoprecipitated from the low Wnt5a secreting cell line UACC903 and the interacting mRNA was measured by quantitative real-time PCR.^40^ Mov10 interaction with Wnt5a, SCD or FASN mRNA transcripts was detected over control IgG immunoprecipitation relative to total input mRNA (Figures 5d–f). These results are consistent with Mov10 regulating Wnt5a, SCD and FASN expression by direct interaction with the mRNA transcript.

### High Mov10 expression is associated with low FASN expression in melanoma tumors

**Figure 6:**
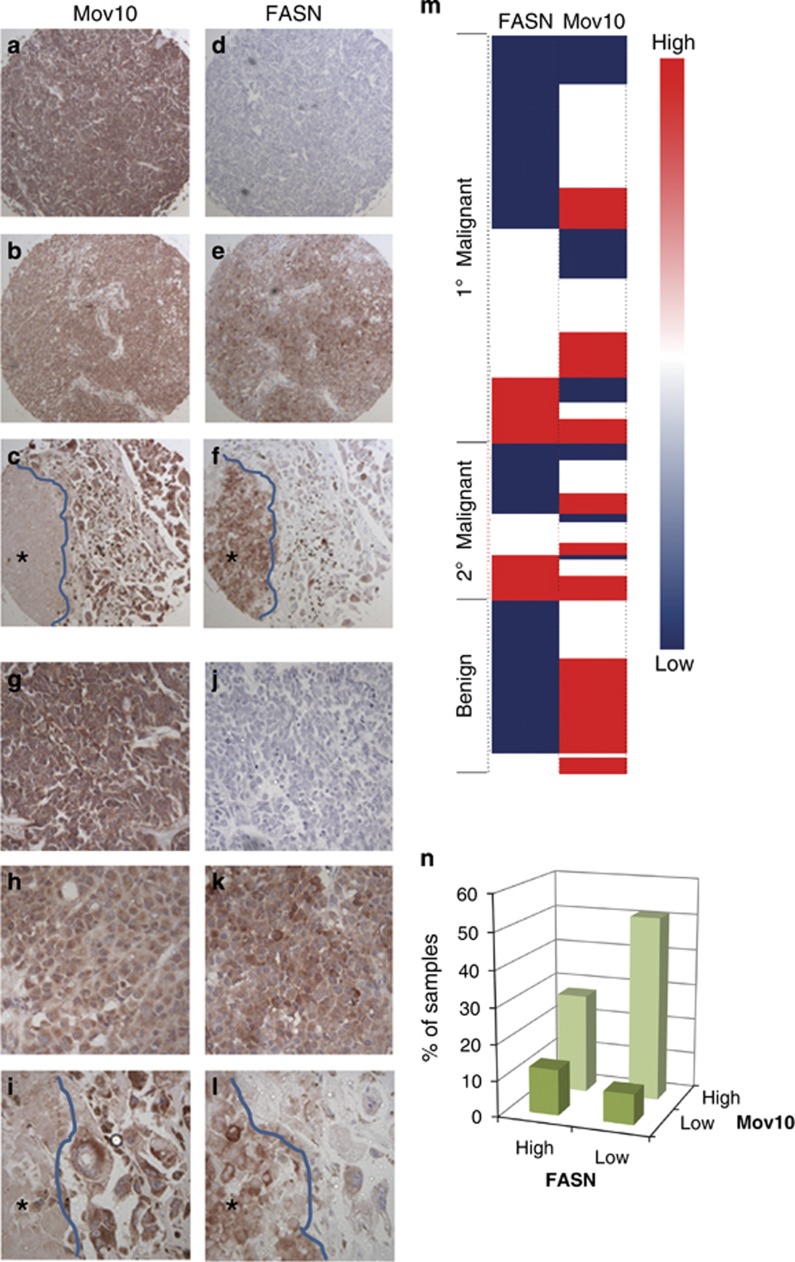
Expression of Mov10 and FASN in melanoma tumors. (**a–c**) Mov10 immunohistochemistry (IHC) staining of benign and malignant tumors at × 5 magnification. (**d**–**f**) Serial sections of tumors in (**a–c**) IHC stained for FASN at × 5 magnification. (**g–l**) Samples shown in (**a–f**) at × 20 magnification (asterisk indicates tumor area outlined in blue in (**c**, **i**, **f** and **l**). (**m**) Samples were blindly scored and divided into three groups according to staining intensity: low (score 1), medium (score 2) and high (score 3). The samples were further stratified by tumor stage. The scores were ranked in each antibody group and the heat map was generated from the ranked scores. Red and blue rectangles represent expression levels greater than or less than the mean (white), respectively (*N*=179). (**n**) Quantitation of high (2+3) and low (1) expression levels of Mov10 and FASN in benign and primary malignant tumors scored in (**m**) as a percent of total samples (Pearson's correlation coefficient *r*=−0.27; *P*<0.01; *N*=141).

Our studies indicate that Mov10 shRNA increases expression of FASN and SCD in cultured melanoma cell lines promoting Wnt5a secretion. We asked whether the expression of FASN or SCD also correlated with Mov10 expression in human melanoma tumors. Tissue microarrays were stained by immunohistochemistry for Mov10 and FASN expression and the intensity of staining was blindly scored. In high-Mov10-expressing tumors, the levels of FASN were frequently lower than in low-Mov10-expressing tumors that generally expressed higher levels of FASN (Figures 6a, d, g, j, c, f, i and l). Some of these tumor samples contained both high- and low-Mov10-expressing regions and the levels of FASN staining in each region was the inverse of the level of Mov10 (Figures 6c, f, i and j). Quantification of Mov10 and FASN levels reveal a negative correlation between Mov10 and FASN protein expression in primary melanoma and benign tumors. We observed high-Mov10-expressing cells are most commonly benign tumors with low FASN expression, whereas low-Mov10-expressing tumors with high FASN expression were more often malignant melanoma. Secondary tumors trended toward higher FASN expression, but Mov10 levels were not biased toward high or low expression (Figure 6m). The tissue arrays did not contain sufficient stage III and IV tumor samples to correlate expression of Mov10 and FASN with tumor stage. When the secondary tumors are excluded, there is a negative correlation between Mov10 and FASN expression levels in melanoma tumors (*r*= −0.27; *P*<0.01; *N*=141) (Figure 6n). This is consistent with the model where Mov10 represses enzymes involved in lipid metabolism.

## Discussion

Although it is known that Wnt ligands require lipid modification for secretion, the regulation of the lipid modification in homeostatic and disease states is not well studied. In melanoma, Wnt5a levels have been shown to correlate with tumor progression, but the function of Wnt5a in the initiation and progression of other cancers is much less clear. In the cell lines we examined the cellular levels of Wnt5a do not correlate with secreted Wnt5a levels in melanoma cell lines. Without knowing whether Wnt5a is secreted it is difficult to determine whether the expression level of Wnt5a in a tumor also indicates the level of Wnt5a signaling activity. Alternatively, the expression of proteins that promote Wnt5a maturation and secretion could provide additional markers to correlate with disease progression.

Inhibition of Mov10 increased the amount of Wnt5a in the conditioned media, but not the cell lysate. The question of whether the increase in secreted Wnt5a is simply because of increased Wnt5a production was addressed by measuring the level of lipid-modified Wnt5a in the cell lysate. Although inhibition of Mov10 did not alter the levels of Wnt5a in the cell lysate, the amount of acylated Wnt5a increased dramatically. As Wnt5a acylation is required for secretion, it is fair to assume that the large increase in Wnt5a acylation caused by Mov10 shRNA also increases the amount of secreted Wnt5a. Furthermore, the increase in secreted Wnt5a did not reduce the levels of cellular Wnt5a, indicating the total level of Wnt5a protein must also be increasing. However, as a transgene lacking the endogenous Wnt5a promoter and 5′ and 3′-UTR sequences also maintains cellular levels of Wnt5a, this is not likely due to regulation of Wnt5a mRNA levels. We think it is more likely that there is a change in trafficking of Wnt5a from a degradative pathway to the secretory pathway upon Mov10 inhibition.

We expected that the increase in secreted Wnt5a in Mov10 shRNA-expressing cells would be caused by increased PORCN expression as Wnt signaling has been shown to be sensitive to PORCN levels.^41^ We did not observe increased PORCN expression with Mov10 shRNA. Instead, we observed increased protein expression of enzymes involved in FASN and the desaturation of palmitoyl-CoA. These data were consistent with previous PAR-CLIP mRNA sequencing studies by Gregersen *et al.*^22^ that showed Mov10 binds FASN and SCD, but not PORCN mRNA transcripts. Our studies confirmed Mov10 shRNA upregulates this lipid synthesis pathway by profiling the fatty acyl-CoA forms by mass spectrometry, revealing a specific increase of palmitoyl-CoA and palmitoleoyl-CoA.

FASN is frequently overexpressed in melanoma and other cancers, yet the molecular events that cause this increase as well as the function of elevated FASN in cancers are still unclear.^33, 34, 35, 36, 37, 38^ It has been suggested that elevated FASN expression promotes cell survival under adverse conditions, but the importance of lipid synthesis in signal transduction is relatively unexplored. Our findings reveal a previously unappreciated role for aberrant lipid biosynthesis that increases Wnt secretion and increased metastatic behavior. Previous studies have shown that overexpression of FASN resulted in increased lipid-modified canonical Wnt3a and stabilized β-catenin, suggesting FASN may be sufficient to also increase production of Wnt3a.^42^ Our results show that inhibitors to FASN effectively inhibited the Wnt5a secretion induced by Mov10 shRNA, demonstrating a functional role for elevated FASN in the increase in secreted Wnt5a. Inhibition of Wnt5a acylation and secretion with inhibitors of FASN and SCD did not cause accumulation of Wnt5a in the cell lysates. This may indicate that when Wnt5a is not lipid modified, it is alternatively trafficked to the lysosome for degradation.

It is unclear whether Mov10 regulates the expression of Wnt5a, FASN and SCD through mRNA stability or by suppressing mRNA translation. The increased level of mRNA may be an indirect effect caused by an increase in translation.^39^ Mov10 binds mRNAs encoding Wnt5a, FASN and SCD, and when Mov10 is inhibited by shRNA, expression of these targets is increased at the mRNA and protein levels. Changes in expression of individual genes by miRNAs are generally relatively small, but targeting multiple points of a pathway is thought to result in a large overall effect on the output of the pathway.^43^ Similarly, inhibition of Mov10 causes relatively small changes in FASN and SCD expression, but dramatically increases Wnt5a acylation, secretion and cell invasion.

Inhibition of Mov10 increases the mRNA and protein levels of multiple regulators of Wnt5a production, increasing both Wnt5a lipid modification and secretion. This mechanism would make Mov10 a key suppressor of Wnt5a secretion in melanoma, the loss of which would lead to increased Wnt5a-mediated invasion. Identifying the molecular changes that decrease Mov10 expression in melanoma warrants further study.

## Materials and Methods

### Antibodies and inhibitors

Anti-Mov10 antibodies (ab80613) were from Abcam (Cambridge, MA, USA). Anti-PORCN antibodies (HPA049215) were from Sigma (St Louis, MO, USA). Anti-FLAG M2 antibodies (F3165) were from Sigma. Anti-HA antibody (MMS-101R) was from Covance (Conshohocken, PA, USA). Anti-Wnt5a (AF645) and anti-Ror2 (AF2064) were from R&D Systems (Minneapolis, MN, USA). Anti-histone H2B, anti-Histone H3, anti-β-actin, anti-β-tubulin and anti-GAPDH antibodies were from Cell Signaling Technologies (Danvers, MA, USA). Anti-FASN (610962) antibodies were from BD Transduction Laboratories (Franklin Lakes, NJ, USA). FASN inhibitor, Cerulenin (C2389), was from Sigma. SCD inhibitor, CAY10566 (10012562), was from Cayman Chemical (Ann Arbor, MI, USA).

### Mov10 shRNA and expression vectors

The coding sequences of Mov10 was amplified from pFLAG/HA-MOV10 (Addgene, Cambridge, MA, USA) by PCR and inserted into pcDNA3.1(hygro+) vector-FLAG or pcDNA3.1(hygro+) vector-FLAG. The coding sequences of Ago2 was amplified from pIRESneo-FLAG/HA Ago2 by PCR and inserted into pcDNA3.1 (hygro+) vector-HA. The coding sequences of Mov10 was amplified from pcDNA3.1-Mov10-FLAG and inserted into the *Age*I/*Sal* site of pRRLSIN.cPPT.PGK-GFP.WPRE. The Mov10 short hairpin-resistant mutations in pRRLSIN-Mov10-R2 were made by using the QuikChange site-directed mutagenesis kit (Agilent Technologies, Stratagene, Santa Clara, CA, USA) according to the manufacturer's protocol, resulting in silent mutation in the shMov10-2 target sequences.

The oligonucleotides for shMov10 and shCtr construct were synthesized (Integrated DNA Technologies, Coralville, IA, USA) and inserted into the pLKO.1 vector. shCtr encodes the nontargeting sequence of SHC002 (Sigma). The shRNA target sequences of human Mov10 are 5′-GCTGACCTTCAAGGTGAACTT-3′ (shMov10-1), 5′-CGTTACTGCATCACCAAACTT-3′ (shMov10-2), 5′-CAGCTGGATCTGGACTTTAAT-3′ (shMov10-4). Cells were transduced with lentivirus encoding shCtr or shMov10 and selected by puromycin treatment (1 μg/ml).

### RNA isolation and quantitative real-time PCR

Total RNA was isolated with TRIZOL (Invitrogen, Carlsbad, CA, USA) following the instructions of the manufacturer. Reverse transcription was performed using oligo dT18-20 primers and SuperScriptIII reverse transcriptase (Invitrogen). Complementary DNA was amplified using SYBR Green 2 × PCR Master Mix (Applied Biosystems, Foster City, CA, USA) with 40 cycles of 15 s denaturation at 94 °C, 15 s annealing at 60 °C and 1 min extension at 72 °C. RPL32 or 18s was used as normalization control. Primer sequences for real-time PCR are listed below: human SCD5 forward 5′-CACTCTGCTCTGGGCCTAC-3′, reverse 5′-CCGTCTCTGAGTACTTGTGGT-3′, human SCD forward 5′-ACTTGGAGCTGTGGGTGAG-3′, reverse 5′- ACTTTCTTCCGGTCATAGGC-3′, human FASN forward 5′-GGCCTACACCCAGAGCTA-3′, reverse 5′- GCCTCCAGCACCCTGTT-3′, human Wnt5a forward 5′-TAAGCCCAGGAGTTGCTTTG-3′, reverse 5′-CAGAGAGGCTGTGCTCCTA-3′, human RPL32 forward 5′-TGTGCAACAAATCTTACTGTGC-3′, reverse 5′-GTGACTCTGATGGCCAGTTG-3′, human PORCN forward 5′-TCAGTGCCTGTGTCTTGTCA-3′, reverse 5′-GTAGCCCTGCTCCTCTGTG-3′, human 18 s forward 5′-AAACGGCTACCACATCCAAG-3′, reverse 5′-CCTCCAATGGATCCTCGTTA-3′.

### Cell culture and transfections

HEK 293T, UACC903, M93-047, FS5 and WM239A cells were cultured in RPMI media containing 10% fetal bovine serum (FBS). All transfections were carried out using TransIT-LT1 (Mirus) according to the manufacturer's instructions.

### Palmitoylation assay

Cell were labeled with 100 μM palmitic acid azide (Life Technologies) for 6 h at 37 °C. Cells were lysed in 200 μl lysis buffer (50 mM Tris, pH 7.5, 1% SDS, 1 μg/ml leupeptin, 1 μg/ml aprotinin and 2 μg/ml pepstatin A). Lysates were sonicated and centrifuged at 15 000 RPM for 10 min. Then, 90 μl of lysate was reacted with biotin alkyne (Life Technologies) using the Click-IT assay (Life Technologies) as per the manufacturer's protocol. Biotinylated proteins were isolated using streptavidin agarose (Pierce, Rockford, IL, USA) and washed 5 times in lysis buffer and analyzed by SDS–polyacrylamide gel electrophoresis and immunoblotting.

### Immunoprecipitation

HEK 293T cells were seeded in six-well plates. Each well of HEK293T cells was transfected with 2.5 μg of plasmids in total. At 60 h after transfection, cells were treated with recombinant Wnt5a protein for 30 min in fresh complete media. Then, cells were washed once with phosphate-buffered saline and lysed by scraping in immunoprecipitation buffer (1% Triton X-100, 50 mM Tris, pH 7.5, 150 mM NaCl, 10 mM NaF, 2 mM NaVO_4_, 1 mM pyrophosphoric acid and protease inhibitors) and incubated at 4 °C for 30 min while rotating. Lysates were centrifuged at 15 000 *g* for 15 min at 4 °C. For FLAG-tag immunoprecipitation, the supernatant was added to 15 μl prewashed anti-FLAG M2 magnetic beads (Sigma) and incubated at 4 °C overnight while rotating. The next day, magnetic beads were washed four times and eluted in 50 μl immunoprecipitation buffer containing 2 μl FLAG-peptide (Sigma, 5 mg/ml) for 1 h on ice.

### Spheroid assay

The 96-well plates were coated with 50 μl per well of sterile 1.5% Noble agar and solidified at room temperature for 10 min. Next, 200 μl of 2.5 × 10^4^ cells/ml cell suspension was added to each well. Spheroids formed at 37 °C and 4.0% CO_2_ for 24 h. Collagen matrix was prepared on ice using Advanced Biomatrix (Carlsbad, CA, USA) Pur Col purified bovine collagen, Hyclone (Logan, UT, USA) RPMI 1640 5 × with sodium bicarbonate diluted to 1 × in total volume and 10% FBS. Sterile NaOH was then added to correct collagen pH. Then, 75 μl of collagen matrix was added to new wells and allowed to solidify at 37 °C for 1 h. Spheroids were resuspended in 125 μl of collagen matrix and transferred to wells containing 75 μl of collagen. After collagen solidified at 37 °C, 100 μl of fresh media was added on top of collagen. Media were changed after day 3. Images were taken every 24 h for 6–7 days.

The highest secreting melanoma cell line FS5 was unable to form spheroids and instead the FS5 cells were green fluorescent protein (GFP) labeled and co-cultured with unlabeled WM239A cells (1:3 ratio). The co-cultures formed spheroids containing both GFP-labeled FS5 cells and unlabeled WM239A cells. The labeled FS5 cells were the most invasive and began invading the collagen by the second day (Figure 1b).

### Ribonucleoprotein immunoprecipitation

The ribonucleoprotein immunoprecipitation was performed as described by Keen *et al.*^40^ Briefly, four 10 cm dishes of WM239A cells were washed once with 5 ml cold phosphate-buffered saline. Then, cells were scraped in phosphate-buffered saline into 15 ml conical tubes. The tubes were centrifuged at 500 *g* for 5 min and cell pellets were resuspended in 80 μl polysome lysis buffer (100 mM KCl, 5 mM MgCl_2_, 10 mM HEPES, pH 7.0, 0.5% NP40, 1 mM DTT, 100 units/ml RNase Out, 400 μM VRC, 10 mM NaF, 2 mM NaVO_4_, 1 mM pyrophosphoric acid and protease inhibitors). The lysates were incubated on ice for 15 min and stored at −80 °C overnight. Protein-A Sepharose beads (20 μl/tube) in NT2 supplemented with 5% bovine serum albumin (BSA) were pre-swollen for at least 1 h at 4 °C before use. Then, 4 μg/tube IgG or Mov10 antibody was added into bead slurry and rotated at 4 °C for 5 h. Antibody-coated beads were washed with 0.5 ml of ice-cold NT2 buffer 3 times before use. The mRNP lysates were thawed on ice, and 1440 μl NT2 buffer (50 mM Tris-HCl, pH 7.4, 150 mM NaCl, 1 mM MgCl_2_, 0.05% NP40, 1 mM DTT, 100 units/ml RNase Out, 400 μM VRC, 10 mM NaF, 2 mM NaVO4, 1 mM pyrophosphoric acid and protease inhibitors) was added into lysates and centrifuged at 15 000 *g* for 15 min. Next, 650 μl clear supernatant was transferred into IgG or Mov10 bead mixture and rotated at 4 °C overnight. Clear supernatant at 50 μl was saved as input. Beads were washed four times with 0.5 ml NT2 buffer and beads were incubated with 100 μl NT2 buffer supplemented with 30 μg proteinase K for 30 min at 55 °C. Trizol reagent (Invitrogen) was added directly to the pellet to release the RNP components and isolate the RNA.

### Isolation of Wnt5a from conditioned media

Melanoma cells were counted and equal amount of cells were plated into 10 cm dishes. Cells were grown in 10 ml RPMI 1640 plus 2 mM Glutamine (minus or plus 10% FBS) for 24 h and the media were collected and centrifuged at 2000 *g* for 10 min at 4 °C. The conditioned media were solubilized with 1% CHAPS for 3 days followed by incubation with 40 μl Blue Sepharose High Performance (90-1001-23, GE Healthcare, Piscataway, NJ, USA) overnight. Beads were washed 1 × with 1 ml wash buffer (1% CHAPS, 150 mM KCl, 20 mM Tris-HCl, pH 7.5) and boiled with 60 μl 5 × loading buffer.

### Immunohistochemistry staining

Human Malignant melanoma, metastatic malignant melanoma and nevus tissue array (ME1004a and ME1004b, US Biomax, Rockville, MD, USA) were used for immunohistochemistry staining. Antigen retrieval was achieved by microwaving arrays immersed in 1 liter Tris-EDTA buffer (10 mM Tris base, 1 mM EDTA solution, 0.05% Tween 20, pH 9.0) for 30 min. Tissue arrays were treated with 1% H_2_O_2_ in water for 15 min and blocked in 10% BSA in Tris-buffered saline for 2 h at room temperature. Anti-Mov10 (1:200) in 10% BSA/Tris-buffered saline or anti-FASN (1:500) in 1% BSA/Tris-buffered saline were applied and incubated at 4 °C overnight. Then, arrays were incubated with biotinylated anti-rabbit (1:500, Vector Lab.) for Mov10 or rabbit anti-mouse (1:200, Vector Lab., Burlingame, CA, USA) for FASN for 1 h at room temperature followed by being incubated with VECTASTAIN ABC kit (PK-4000, Vector Lab.) for 30 min. Mov10 and FANS were visualized by Vector Novared substrate kit (SK-4800, Vector Lab.). The arrays were counterstained with hematoxylin for 1.5 min.

### Liquid chromatography/mass spectrometry reagents

Ammonium formate solution, 5-sulfosalicilic acid, glacial acetic acid, dimethyl sulfoxide, *N*,*N*-diisopropylethylamine (DIPEA) and pentafluorobenzyl bromide (PFB) were purchased from Sigma-Aldrich (St Louis, MO, USA). Optima liquid chromatography/mass spectrometry grade methanol, acetonitrile (ACN), 2-propanol (IPA) and water were purchased from Fisher Scientific (Pittsburgh, PA, USA). 2-(2-pyridyl) ethyl functionalized silica gel 100 mg/1 ml tubes were obtained from Supelco Analytical (Bellefonte, PA, USA). Streptomycin and penicillin were purchased from Invitrogen. Charcoal-stripped FBS was from Gemini Bioproducts (West Sacramento, CA, USA). [^13^C_3_^15^N_1_]-calcium pantothenate was purchased from Isosciences (King of Prussia, PA, USA).

### Isolation of SILEC standards

Stable isotope labeling by essential nutrients in cell culture (SIlEC) labeling was conducted as previously reported.^44^ Briefly, Hepa1c1c7 cells were passaged at least 7 times in custom RPMI 1640 media without calcium pantothenate and containing 10% charcoal-stripped FBS, 100 units/ml penicillin, 100 mg/l streptomycin and 2 mg/l [^13^C_3_^15^N_1_]-calcium pantothenate. At 24 h before extraction, ‘ultra-labeling' with media as above, but omitting charcoal-stripped FBS, was performed in order to ensure optimal CoA stable isotope labeling. Cells were incubated with 100 μM palmitic acid in 0.025% dimethyl sulfoxide for 3 h to increase levels of long-chain acyl-CoAs.^32^ Upon completion of labeling, Hepa1c1c7 cells were gently lifted, centrifuged at 500 *g* for 5 min and resuspended in 750 μl/10 cm^2^ plate of ACN/IPA (3:1; v/v). Samples were sonicated with a probe tip sonicator on ice 30 times for 0.5 s. Then, 250 μl of 100 mM KH_2_PO_4_ was added to the samples, vortex mixed and spun down for 10 min at 16 000 *g* at 4 °C. The supernatant was pooled and stored in −80 °C until use.

### Acyl-CoA analysis

Cells were gently lifted, centrifuged at 500 *g* for 5 min and resuspended in 850 μl/10 cm^2^ plate of ACN/IPA (3:1; v/v) for extraction as described previously.^32, 45^

For fatty acid analysis below, 100 μl was diverted. The SILEC standard was added (150 μl) and samples were pulse sonicated with a probe tip sonicator on ice 30 times for 0.5 s. Then, 250 μl of 100 mM KH_2_PO_4_ was added to the samples, vortex mixed and spun down for 10 min at 16 000 *g* at 4 °C. The supernatant was transferred to a glass tube and acidified with 125 μl of glacial acetic acid. SPE columns were equilibrated with 1 ml of ACN/IPA/H_2_O/acetic acid (9:3:4:4; v/v) washing solvent. Samples were transferred to the columns that were washed two times with 1 ml of the washing solvent. The acyl-CoAs were then eluted by washing the columns twice with 500 μl methanol/250 mM ammonium formate (4:1; v/v) into glass tubes. After evaporation to dryness under nitrogen gas, the eluates were redissolved in 50 μl of H_2_O/ACN (70:30; v/v) containing 5% 5-sulfosalicilic acid (w/v) and transferred to high-performance liquid chromatography (HPLC) vials ready for liquid chromatography/mass spectrometry analysis.

Samples were kept at 4 °C in a Leap CTC autosampler (CTC Analytics, Zwingen, Switzerland) with 20 μl injections used for liquid chromatography/mass spectrometry analysis. Chromatographic separation was performed using reversed-phase Waters (Milford, MA, USA) XBridge C18 column (2.1 × 150 mm, pore size 3 μm) on an Agilent (Santa Clara, CA, USA) 1100 HPLC system using a three-solvent system: (1) 5 mM ammonium acetate in water, (2) 5 mM ammonium acetate in 95/5 acetonitrile/water (v/v) and (3) 80/20/0.1 (v/v/v) acetonitrile/water/formic acid, with a constant flow rate of 0.2 ml/min. Gradient elution was performed as follows: 2% B (isocratic) for 1.5 min, 2% to 20% (linear gradient) over 3.5 min, 20–100% B (linear gradient) B over 0.5 min, 100% B (isocratic) for 8 min and 100% C for 5 min, before equilibration at initial conditions for 5 min.

Samples were analyzed using an API 4000 triple quadrupole mass spectrometer (Applied Biosystems) in positive electrospray ionization mode and data were analyzed using Analyst software (SCIEX, Framingham, MA, USA) as described previously.^32, 46, 47^ Mass spectrometer operating conditions were as follows: ion spray voltage (5.0 kV), compressed air as curtain gas (15 psi) and nitrogen as nebulizing gas (8 psi), heater (15 psi) and collision-induced dissociation gas (5 psi). The electrospray ionization probe temperature was 450 °C, the declustering potential was 105 V, the entrance potential was 10 V, the collision energy was 45 eV and the collision exit potential was 15 V. CoA thioesters were monitored using transitions corresponding to the neutral loss of 507 amu from the parent ion.

## Figures and Tables

**Table 1:** Quantitation of Mov10 immunohistochemistry (IHC) staining shown in Figure 1c

*Tumor*	*+++*	*++*	*+*	N
Nevus	66.6%	33.3%	0%	24
Primary	10.7%	44.7%	44.7%	47
Metastatic	33.3%	33.3%	33.3%	15
