# Supplementary material for: Regulation of lipid synthesis by the RNA helicase Mov10 controls Wnt5a production
Source: Oncogenesis. 2015 Jun 1;4(6):e154–. doi: 10.1038/oncsis.2015.15 (PMC4753523; doi:10.1038/oncsis.2015.15)
Supplement: Supplementary Information [file oncsis201515x1.pdf]

## Supplementary Figure Legend

### Supp. Figure S1

Wnt5a mRNA levels relative to L32 mRNA. Error bars denote standard deviation.

### Supp. Figure S2

Inhibition of Ror2 protein expression with two shRNA constructs.

### Supp. Figure S3

PORCN protein expression in UACC903 cells expressing control shRNA, PORCN shRNA #1, 2, 3. Tubulin is a loading control.

### Supp. Figure S4

Collagen invasion assay of control and Mov10 shRNA expressing cells treated with the FASN inhibitor cerulenin.
