# Supplementary figures and images for: Regulation of lipid synthesis by the RNA helicase Mov10 controls Wnt5a production
Source: Oncogenesis. 2015 Jun 1;4(6):e154–. doi: 10.1038/oncsis.2015.15 (PMC4753523; doi:10.1038/oncsis.2015.15)

Wang et al. Supp. Figure S1

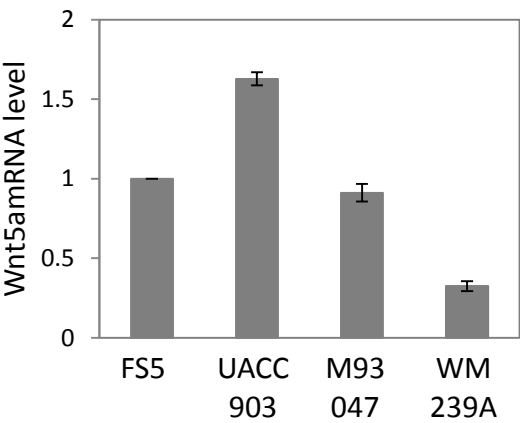

Wang et al. Supp. Figure S2

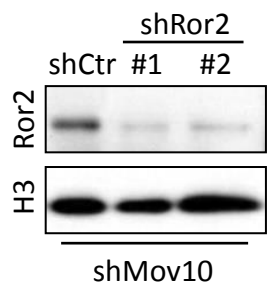

Wang et al. Supp. Figure S3

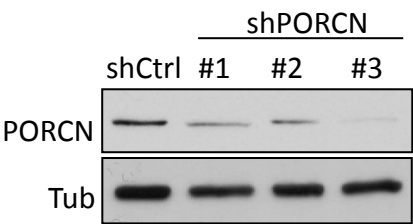

Wang et al. Supp. Figure S4

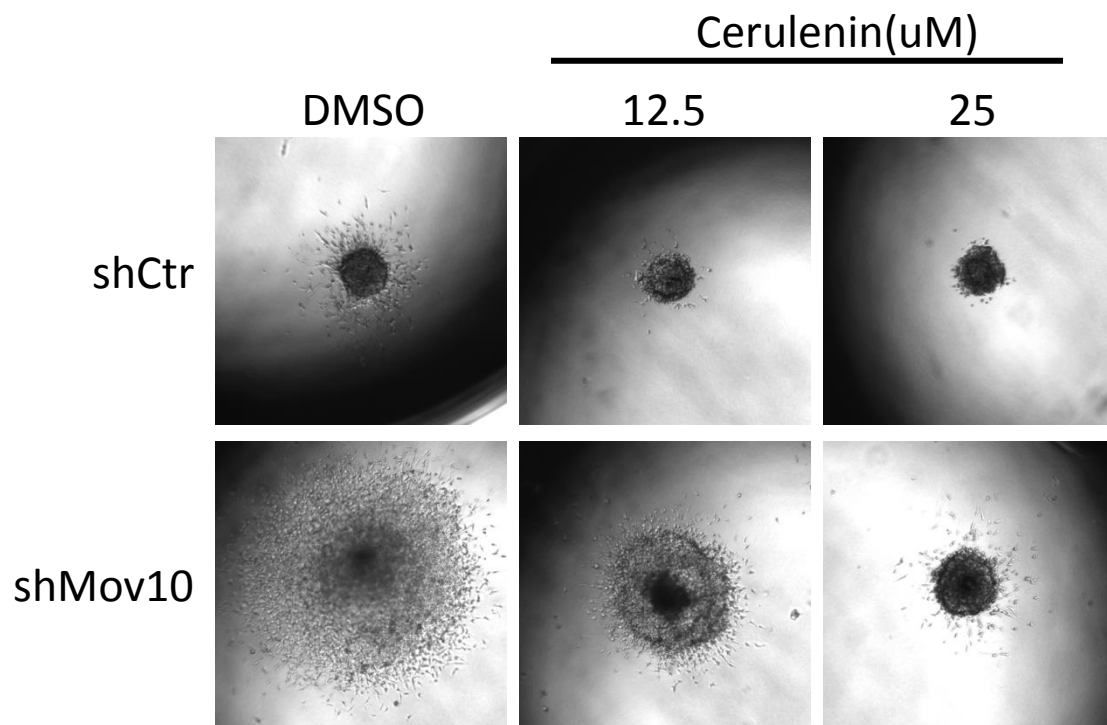

Supplement: Supplementary Figures [file oncsis201515x2.pdf]
